# Transcriptional Response to Lactic Acid Stress in the Hybrid Yeast Zygosaccharomyces parabailii

**DOI:** 10.1128/AEM.02294-17

**Published:** 2018-02-14

**Authors:** Raúl A. Ortiz-Merino, Nurzhan Kuanyshev, Kevin P. Byrne, Javier A. Varela, John P. Morrissey, Danilo Porro, Kenneth H. Wolfe, Paola Branduardi

**Affiliations:** a UCD Conway Institute, School of Medicine, University College Dublin, Dublin, Ireland; b Department of Biotechnology and Biosciences, University of Milano-Bicocca, Milan, Italy; c School of Microbiology, University College Cork, Cork, Ireland; d Centre for Synthetic Biology and Biotechnology, University College Cork, Cork, Ireland; e Environmental Research Institute, University College Cork, Cork, Ireland; f APC Microbiome Institute, University College Cork, Cork, Ireland; University of Tennessee and Oak Ridge National Laboratory

**Keywords:** RNA-seq, Zygosaccharomyces parabailii, hybrid, lactic acid, stress response, yeasts

## Abstract

Lactic acid has a wide range of applications starting from its undissociated form, and its production using cell factories requires stress-tolerant microbial hosts. The interspecies hybrid yeast Zygosaccharomyces parabailii has great potential to be exploited as a novel host for lactic acid production, due to high organic acid tolerance at low pH and a fermentative metabolism with a high growth rate. Here we used mRNA sequencing (RNA-seq) to analyze Z. parabailii's transcriptional response to lactic acid added exogenously, and we explore the biological mechanisms involved in tolerance. Z. parabailii contains two homeologous copies of most genes. Under lactic acid stress, the two genes in each homeolog pair tend to diverge in expression to a significantly greater extent than under control conditions, indicating that stress tolerance is facilitated by interactions between the two gene sets in the hybrid. Lactic acid induces downregulation of genes related to cell wall and plasma membrane functions, possibly altering the rate of diffusion of lactic acid into cells. Genes related to iron transport and redox processes were upregulated, suggesting an important role for respiratory functions and oxidative stress defense. We found differences in the expression profiles of genes putatively regulated by Haa1 and Aft1/Aft2, previously described as lactic acid responsive in Saccharomyces cerevisiae. Furthermore, formate dehydrogenase (*FDH*) genes form a lactic acid-responsive gene family that has been specifically amplified in Z. parabailii in comparison to other closely related species. Our study provides a useful starting point for the engineering of Z. parabailii as a host for lactic acid production.

**IMPORTANCE** Hybrid yeasts are important in biotechnology because of their tolerance to harsh industrial conditions. The molecular mechanisms of tolerance can be studied by analyzing differential gene expression under conditions of interest and relating gene expression patterns to protein functions. However, hybrid organisms present a challenge to the standard use of mRNA sequencing (RNA-seq) to study transcriptional responses to stress, because their genomes contain two similar copies of almost every gene. Here we used stringent mapping methods and a high-quality genome sequence to study the transcriptional response to lactic acid stress in Zygosaccharomyces parabailii ATCC 60483, a natural interspecies hybrid yeast that contains two complete subgenomes that are approximately 7% divergent in sequence. Beyond the insights we gained into lactic acid tolerance in this study, the methods we developed will be broadly applicable to other yeast hybrid strains.

## INTRODUCTION

Species belonging to the *Zygosaccharomyces bailii sensu lato* clade have a remarkable resilience against stress induced by weak acids, some of which are widely used as food preservatives or are versatile chemical platforms (1, 2). Therefore, on the one hand, these yeasts represent a challenging problem in the food industry because they are often found as contaminants in production pipelines for wine, high-sugar products, and canned foods. On the other hand, they are promising cell factories for biotechnological applications involving organic acids that can be produced by microbial fermentation (3, 4) or released by lignocellulosic pretreatment of biomass (5).

Lactic acid is one of the useful organic acids that can be produced by yeasts as a microbial factory. This compound has a wide range of industrial applications, including food preservation, additives, and pharmaceuticals (6), and the potential to be used for bioplastic production from a renewable source (7). Natural fermentation by lactic acid bacteria has long been the main source of industrial lactic acid production (8). Despite important progress using lactic acid bacteria (9), some of these organisms have complex nutritional requirements posing a negative impact on cost effectiveness and on product purity (10). Moreover, operational costs are also increased by the need to convert lactate to lactic acid, which is not the case with engineered yeast cells cultivated at pH well below the pK_a_ of lactic acid (3.78) (7, 11). The production of several weak organic acids, including lactic acid, has reached the industrial scale (4), but there is still room for further production improvement by enhancing production host robustness and/or exploiting novel microbial hosts. Therefore, understanding the mechanism of weak acid tolerance in non-Saccharomyces yeasts such as Zygosaccharomyces is important for the future development of ultraefficient production platforms in which these yeasts are genetically engineered to produce lactic acid.

The mechanisms of weak acid stress tolerance and response have been studied extensively in the model yeast Saccharomyces cerevisiae (12–16). However, this knowledge is far from complete and cannot be applied easily to non-Saccharomyces species. Previous research on tolerance to weak organic acids revealed the capability of *Z. bailii sensu lato* to catabolize acetic and benzoic acids even in the presence of glucose (17, 18). In addition, different Z. bailii strains display specific adaptation traits such as the ability to modulate their cell wall and membrane composition in order to decrease the influx of weak acids (19, 20).

Importantly, the *Z. bailii sensu lato* clade is characterized by substantial genetic diversity. Some strains that were previously considered to be Z. bailii were reclassified in 2013 into two new species called Zygosaccharomyces parabailii and Zygosaccharomyces pseudobailii (21). The name *Z. bailii sensu lato* is used to refer to the species complex that includes these two new species as well as other strains that were not reclassified (Z. bailii sensu stricto). The widely studied strains CLIB213^T^ and IST302 are *Z. bailii sensu stricto* (22, 23). The strains ATCC 60483 (used in this study) and ISA1307 are Z. parabailii, which is a hybrid that was formed naturally by mating between *Z. bailii sensu stricto* and an unidentified Zygosaccharomyces species (24, 25). Z. parabailii genomes contain two copies of almost every gene, differing by 7% in nucleotide sequence on average (25). These genes are referred to as homeologs because they are derived from different organisms; homeologs, or homoeologs, are a particular type of paralog (duplicated gene) (26).

We are exploring the possibility of using Z. bailii sensu lato species as alternative yeast hosts for lactic acid production. We focused on Z. parabailii strain ATCC 60483 because our previous work demonstrated its high tolerance to lactic acid at low pH, characterized by growth without any detectable lag phase or acid consumption (20), under microaerobic conditions. These natural characteristics are promising in terms of possible exploitation for organic acid production, and the potential to develop commercial strains will be enhanced when the molecular basis of its unusual tolerance to low pH, high inhibitor concentrations, and other traits of interest are clarified. As a preliminary step toward metabolic engineering, in this study we sought to investigate the molecular mechanisms of lactic acid tolerance in ATCC 60483 by means of mRNA sequencing (RNA-seq). In general, we found that the Z. parabailii transcriptome responds to lactic acid stress by inducing genes related to oxidative stress response and iron homeostasis in a different way than S. cerevisiae does. In addition, Z. parabailii modulates the transcription of genes related to the cell wall, in agreement with our previous data.

## RESULTS

### Transcriptional profile of Z. parabailii homeolog pairs and duplicated genes in lactic acid stress.

In our previous study, we found that Z. parabailii ATCC 60483, when treated with 40 g liter^−1^ of lactic acid during microaerobic fermentation of glucose, neither consumes the lactic acid nor exhibits a reduction in cell viability, although we could observe acid-induced phenotypic and morphological changes (20). During the fermentation, both control and lactic acid-treated cells consumed glucose and produced ethanol as the main metabolite. No other fermentation metabolites were detected at the end of fermentation. The cells treated with lactic acid showed a 25% reduction in growth rate and a 15% reduction in final biomass titer. In addition, the specific glucose consumption rate was 13% lower than that under a control condition. The yields of ethanol at the end of fermentation were similar under both conditions (20). Our aim here was to study the transcriptional response of Z. parabailii when exposed to a high concentration of lactic acid.

**FIG 1:**
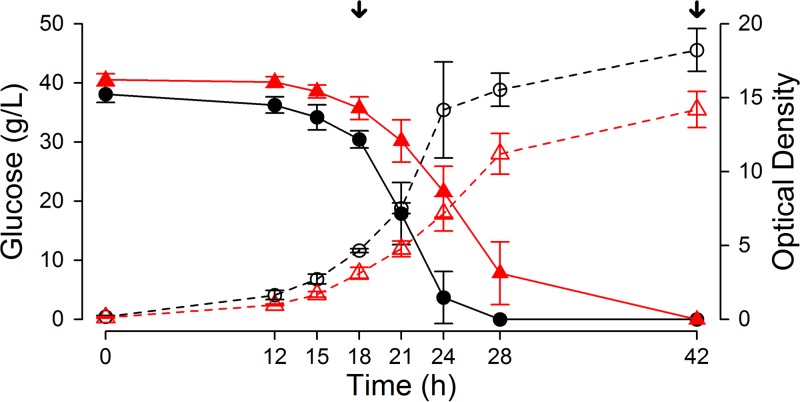
Z. parabailii fermentation profile. Batch bioreactor fermentation was performed in Verduyn medium at pH 3 with the addition of 40 g liter^−1^ lactic acid (red lines) or without lactic acid (black lines). The samples for RNA sequencing were taken at 18 h and 42 h (indicated by arrows), corresponding to exponential phase and postdiauxic shift. Solid lines represent the glucose consumption over time, while dashed lines represent the corresponding OD_660_ values.

We compared the transcriptomes of Z. parabailii ATCC 60483 cultures grown in the presence and absence of lactic acid (40 g liter^−1^), at time points (18 h and 42 h) specifically chosen to ascertain comparable growth kinetics and exclude growth phase-related bias (Fig. 1). After normalizing and filtering the raw RNA-seq read counts, we detected expression for >95% of the Z. parabailii genes under at least one condition, including 36 genes that were transcribed only in lactic acid and 31 that were transcribed only under control conditions (Table 1; see Tables S1 and S2 in the supplemental material).

**TABLE 1:** General overview of the Z. parabailii transcriptional profile^*a*^

Category	No. of genes			
	Control condition	Lactic acid condition	Intersect	Union
Expressed	9,647	9,652	9,616	9,683
Condition specific	31	36	0	67
No evidence of expression^*b*^	438	433	402	469

a The numbers of genes in each category of expression are shown for each condition. After the RNA-seq counts were filtered and normalized, genes were categorized based on their expression profiles under both conditions, with the data pooled from the two time points. Genes showing condition-specific expression only under control conditions, or only in lactic acid, are listed in Tables S1 and S2 in the supplemental material.

b This category includes genes that were discarded by the filtering procedure and genes with no read counts.

**FIG 2:**
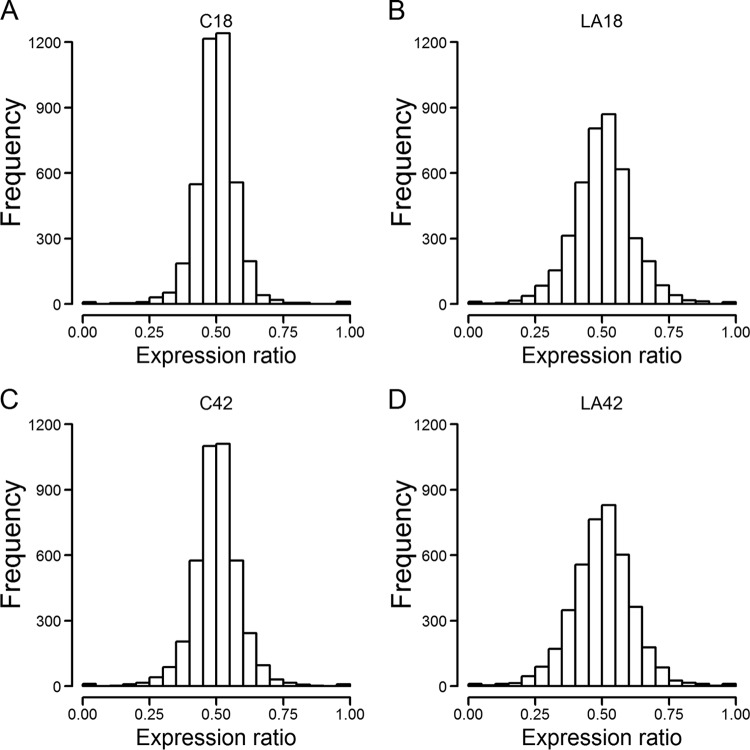
Expression ratios in 4,136 homeologous gene pairs. The expression ratio is defined as A/(A+B), where A and B are the RPKM values (reads per kilobase of mRNA per million transcripts) of the A and B homeologous genes, respectively, averaged among replicates. Histograms show the distribution of expression ratio values under control conditions at 18 h (A), in lactic acid at 18 h (B), under control conditions at 42 h (C), and in lactic acid at 42 h (D).

We used stringent mapping of RNA-seq reads to the genome (see Materials and Methods), in order to capture expression differences between homeologous gene pairs even when they are highly similar in sequence. About 82% of the 10,072 genes in the Z. parabailii nuclear genome show the pattern characteristic of hybrid genomes, forming pairs of A and B homeologs, where the A gene came from one parent in the hybridization and the B gene came from the other (25). Most of the remaining loci in the genome are also present in two copies, but are either A:A or B:B pairs due to loss of heterozygosity after hybridization (25). We calculated the ratio of expression between the A and B homeologs for each of 4,136 A:B gene pairs as described in Materials and Methods (Fig. 2). All but 21 gene pairs showed evidence of expression of both homeologs.

Strikingly, the distribution of expression ratios is broader under lactic acid stress than under control conditions, at both time points. In other words, under the stress condition, one of the two genes in each homeolog pair tends to become predominantly expressed. If we define unbalanced expression as an expression ratio that lies outside the range of 0.4 to 0.6 (Fig. 2), the proportion of homeolog pairs with unbalanced expression is 13.8 to 18.7% under the control conditions but increases to 31.0 to 33.4% under lactic acid conditions. The difference in variance of expression ratios is statistically significant (Fligner-Killeen test; *P* = 3e−98 at 18 h, and *P* = 9e−61 at 42 h).

The distribution of expression ratios is approximately symmetrical (Fig. 2), indicating that in some homeolog pairs the A gene is more highly expressed than the B gene (ratios > 0.5), whereas in others the B gene is more highly expressed (ratios < 0.5). The A genes were derived from the parental species Z. bailii in the hybridization, and the B genes were derived from the other parent (an unidentified Zygosaccharomyces species) (25). Thus, broadly speaking, the cell responds to lactic acid stress by inducing greater divergence of expression between the genes in a homeolog pair, without a strong preference as to whether the A gene or the B gene is the more highly expressed one. There is a trend toward higher expression of the A genes, as illustrated by the larger numbers of loci for which the expression level of the A gene exceeds that of the B gene, as opposed to the converse (Table 2, Ab and aB columns). Statistical analysis indicates a weak bias toward A genes, showing slight but consistent negative skew values, but this bias is significant only in the cells treated with lactic acid for 18 h (Table 2). In summary, Z. parabailii has a slight tendency to express its A genes more highly than its B genes, and this tendency is maintained under lactic acid stress, but the magnitude of this tendency is small compared to the grossly increased divergence of expression levels between homeologs that occurs under lactic acid stress.

**TABLE 2:** Expression ratio between homeologous gene pairs

Group^*a*^	Expression ratio^*b*^				No. of homeolog pairs^*c*^		*P* value^*d*^	% of homeolog pairs with unbalanced expression^*e*^
	Mean	Median	SD	Skew	Ab	aB		
C18	0.500	0.500	0.079	−0.045	2,077	2,059	1	13.8
LA18	0.503	0.505	0.113	−0.009	2,151	1,985	0.041	31.0
C42	0.501	0.501	0.089	−0.146	2,088	2,048	1	18.7
LA42	0.500	0.503	0.115	−0.085	2,119	2,017	0.465	33.4

a C18, control condition at 18 h; LA18, lactic acid condition at 18 h; C42, control condition at 42 h; LA42, lactic acid condition at 42 h.

b Mean, median, standard deviation (SD), and skew refer to the A/(A+B) expression ratios for 4,136 homeologous gene pairs, as described in Materials and Methods.

c Ab, homeolog pair in which the A gene shows higher expression; aB, homeolog pair in which the B gene shows higher expression.

d *P* values refer to the comparison between the number of Ab homeolog pairs and the number of aB homeolog pairs under each of the four conditions. *P* values were obtained from two-sided exact binomial tests of the null hypothesis that the numbers of Ab and aB loci are equal and were corrected for multiple testing using the Bonferroni method.

e Expression ratio values of ≤0.4 and ≥0.6 are categorized as unbalanced expression.

Our method of high-stringency mapping of RNA-seq reads to a high-quality genome sequence detected transcriptional profiles of homeologous gene pairs even where the gene pairs were highly similar. Nevertheless, we needed to modify it to determine read counts for genes that occur in identical pairs (see Materials and Methods). Specifically, among the 402 genes that have no evidence of expression in the “full” count data set, 42% are genes that were affected by loss of heterozygosity (genes in A:A pairs or B:B pairs). The modified method enabled us to measure the combined expression of 230 duplicated genes in Z. parabailii, including the two orthologs of the S. cerevisiae major mitochondrial d-lactate dehydrogenase gene *DLD1* (*I04780*_*A* and *N05010*_*A*, which are identical) and the minor isoform gene *DLD2* (*B01190*_*N* and *G05430*_*N*, which are identical). Although we cannot investigate the expression of each of these identical gene pairs individually, their RPKM (reads per kilobase of transcript per million mapped reads) values are low compared with those of all duplicated genes under lactic acid conditions. For example, the combined RPKM values under lactic acid conditions at 18 h for the two *DLD1* genes was 340.2, and for the two *DLD2* genes the combined value was 710.5, while the average RPKM values for duplicated genes under this condition and at this time point was 4,028. Furthermore, *DLD1* shows a statistically significant 2-fold decrease in expression in lactic acid at both time points, whereas *DLD2* shows no significant expression changes (Data Set S4). These observations are consistent with the previously reported lack of lactic acid consumption of *Z. parabailii* under microaerobic growth (20).

### Upregulated genes are related to oxidation-reduction processes and ion transport in the mitochondria.

**FIG 3:**
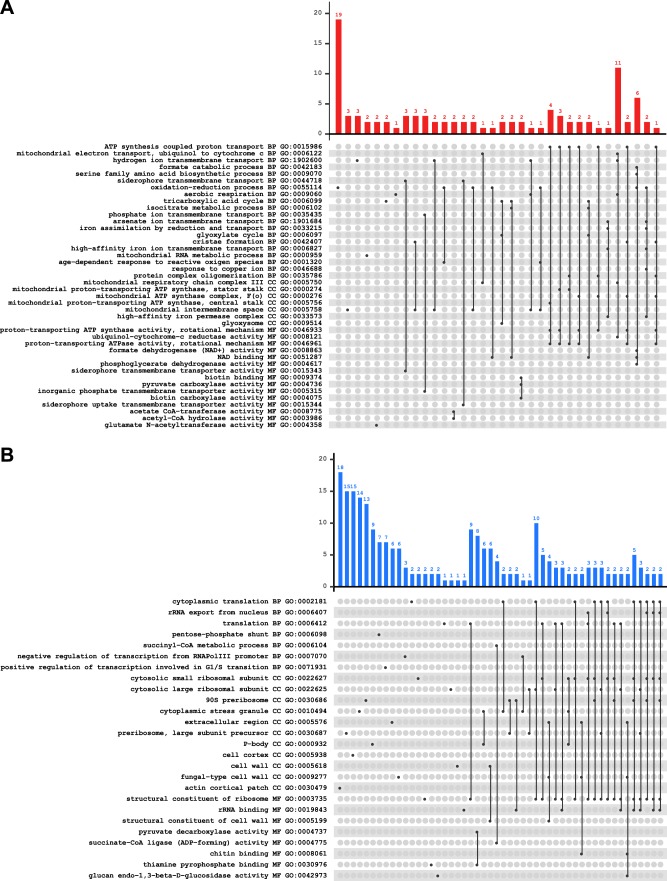
Enriched GO terms among differentially expressed genes. Bar plots show the numbers of differentially expressed genes associated with a GO term (dots) or with a group of GO terms (dots connected by vertical lines). (A) Upregulated genes; (B) downregulated genes. For example, among the 33 upregulated genes with the term GO:0055114 for the oxidation-reduction process in panel A, 19 show only this term, 2 also show the term GO:0001320 for age-dependent response to reactive oxygen species, and so forth. The GO terms are ordered by ontology type (BP, biological process; CC, cellular component; MF, molecular function) and by decreasing adjusted *P* value, always <0.05 (values are in Data Set S6 in the supplemental material).

The “full” set of counts (see Materials and Methods) for the 9,683 genes in the union set of expressed genes (Table 1) were then used for differential expression analysis, with the results filtered for an adjusted *P* value of <0.05 and a ∣log_2_-fold change∣ of ≥1 (Data Set S5). This analysis is independent from that of the duplicated genes mentioned above and identified a total of 227 genes upregulated in lactic acid, of which 117 are specific to 18 h and 83 are specific to 42 h (Table 3). Similarly, a total of 1,019 downregulated genes were found, including 430 specific to 18 h and 431 specific to 42 h. We then performed a gene ontology (GO) term enrichment analysis to identify GO terms that were enriched at both time points in either the upregulated genes (Fig. 3A) or the downregulated genes (Fig. 3B).

**TABLE 3:** Z. parabailii differential expression analysis^*a*^

Category	No. of genes			
	18-h specific	42-h specific	At both time points	At either time point
Upregulated	117	83	27	227
Downregulated	430	431	158	1,019

a The data for the upregulated and downregulated gene categories are the numbers of genes with an adjusted *P* value of <0.05 and a log_2_-fold change of ≥1 or a log_2_-fold change of ≤1, respectively, between lactic acid and control conditions. The sets of genes were further classified into those with altered expression only at 18 h, only at 42 h, at both time points, or at either time point.

When we use an S. cerevisiae gene name in the following functional analysis, we refer to either one or both of its orthologs in a Z. parabailii homeolog pair. These genes are included in our functional analysis if at least one of the members of a Z. parabailii homeolog pair was differentially expressed.

The enriched GO term associated with the highest number of genes upregulated by lactic acid in our data set is GO:0055114 for “oxidation-reduction process” (Fig. 3A; Data Set S6). This term is associated with 33 genes, including homologs of the S. cerevisiae genes *GOR1*, *AIM17*, *CCP1*, *MET13*, *SOD2*, *SOD1*, *GND1*/*GND2*, and *GRX1*/*GRX2*, some of which are also related to enriched mitochondrial terms (GO:0005758, for example). We also observed enrichment for genes in the glyoxylate cycle (GO:0006097) and the glyoxysome (GO:0009514), including homologs of *ICL1* and *IDP2*. The upregulation of these genes along with *FBP1* could indicate activation of the anaplerotic reactions, probably caused by oxygen limitation. *ACH1* with coenzyme A (CoA) transferase activity and Z. parabailii gene *L05300_N* (predicted to have epoxide hydrolase activity) were upregulated at both time points and are presumably involved in the enzymatic detoxification process.

The siderophore transmembrane transport term (GO:0044718) was also found enriched in upregulated genes. Genes in this category are members of the MFS_1 family of transporters, potentially involved in iron retention and/or transport (genes *A10040_B*, *B02380*_*A*, *G04250*_*B*, *I00120*_*N*, *I05800*_*A*, and *O00120*_*N*), and upregulated at 18 h. These genes are all classified as integral components of the membrane. Other genes specifically upregulated at 42 h include *FIT2*, *STL1*, and *K05040*_*N*, which shows no sequence homology to S. cerevisiae genes but is predicted to be a transmembrane transporter (see Materials and Methods).

### Downregulated genes are related mainly to components of the cell boundaries and protein translation.

The GO term enrichment analysis for downregulated genes showed 63 genes related to ribosomal functions (GO:0003735) and 38 to cytoplasmic translation (GO:0002181) (Fig. 3B; Data Set S6). Most of those genes were downregulated at 42 h, implying a general decrease in protein synthesis. This response seems to correspond to a general mechanism observed also in other yeasts used as cell factories, e.g., S. cerevisiae under stress conditions (27) and Komagataella phaffii (Pichia pastoris) used for heterologous protein production induced by methanol (28) during stress, possibly related to resilience or energy maintenance. Some of these genes are also related to the enriched terms GO:0000932 and GO:0010494 for P-body and cytoplasmic stress granules involved in mRNA translation and turnover during different stress conditions in S. cerevisiae (29). These categories are downregulated at 18 h in lactic acid-treated cells. One of the components of stress granules that is also downregulated at 42 h codes for a homolog of S. cerevisiae Pab1, the major poly(A) binding protein which has been demonstrated to promote the formation of stress granules (30). A recent study conducted in S. cerevisiae reported that stress granules are not formed in lactic acid-treated cells (31), and a similar situation might also be true for Z. parabailii.

Among the downregulated genes, we also identified many with functions that we summarize as being related to the boundaries of the cell, i.e., to the cell wall and the plasma membrane. The GO terms in this group include the actin cortical patch (GO:0030479), cell cortex (GO:0005938), extracellular region (GO:0005576), fungal-type cell wall (GO:0009277), and structural constituents of the cell wall (GO:0005199) (Fig. 3B). Consistent with this, we noticed enrichment of the GO terms for glucan endo-1,3-beta-d-glucosidase activity (GO:0042973) and chitin binding (GO:0008061). These observations indicate that the cell wall is modulated upon lactic acid stress, in agreement with our previous findings (20). Other genes downregulated at 42 h predicted to be integral components of the membrane are *H01670*_*B* with unknown function, *OPT1*, and *HBT1*. We also found downregulation of *CWP1*, a cell wall protein homolog, and *LDS2*, which is involved in the assembly of the S. cerevisiae spore wall.

### Involvement of Haa1 and Aft1/Aft2-regulated genes in lactic acid stress response.

Previous studies on lactic acid stress response mechanisms in S. cerevisiae indicated an important role of the transcription factors Haa1 and Aft1/Aft2 (32, 33). Therefore, we extracted all the S. cerevisiae genes reported to be targets of either Haa1 or Aft1/Aft2 in YEASTRACT (34), in addition to those identified as lactic acid responsive (32). We then tested whether the Z. parabailii orthologs of these S. cerevisiae genes are differentially expressed in our data set. In this case we ignored the log_2_-fold change cutoff to enable detection of small but still significant changes. The results are shown in Data Set S7.

**FIG 4:**
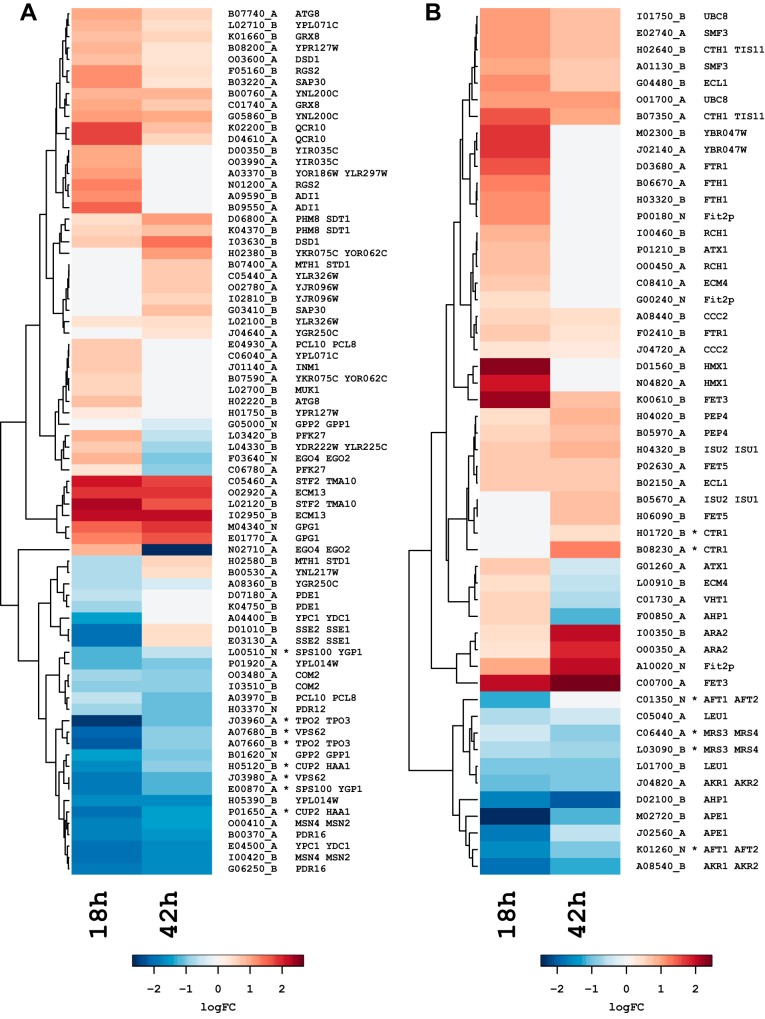
Log_2_-fold changes for Z. parabailii genes putatively controlled by the Haa1 or the Aft1/Aft2 transcription factors. (A) Genes under Haa1 control; (B) genes controlled by Aft1/Aft2. Asterisks indicate S. cerevisiae genes reported as lactic acid responsive by Abbott et al. (32), whose Z. parabailii homologs display an opposite response profile (i.e., upregulated in S. cerevisiae and downregulated in Z. parabailii). Positive log_2_-fold change values in lactic acid versus the control are colored in red as a sign of upregulation, whereas negative values are blue. All the changes shown have an adjusted *P* value of <0.05 (values are in Data Set S7).

We found differential expression of 42 orthologs of S. cerevisiae genes putatively controlled by Haa1 (Fig. 4A). These include the membrane-bound and major weak acid response genes *YPC1/YDC1*, *TPO2/TPO3*, *VPS62/TDA6*, *PDR16*, and *PDR12*. This set also includes the transcription factors *MSN4/MSN2*, *COM2*, and *HAA1/CUP2*, the last of which codes for Haa1 itself. Interestingly, we observed the major weak acid stress response genes *TPO2/TPO3* and *SPS100/YGP1* to be downregulated in Z. parabailii, although they are upregulated during lactic acid stress in S. cerevisiae (32). Here we also found *PFK27* with a response changing from upregulated at 18 h to downregulated at 42 h, and *MTH1/STD1* going from downregulated at 18 h to upregulated at 42 h. These changes in glucose-responsive genes may possibly reflect the diauxic shift.

We performed a similar search strategy for genes putatively under the control of Aft1/Aft2 which in S. cerevisiae are related to iron utilization and homeostasis (35). Results are shown in Fig. 4B and Data Set S7. This strategy detected changes in expression of orthologs of 27 S. cerevisiae genes, of which 6 are downregulated at both time points: *AFT1/AFT2*, coding for the transcription factor itself; *AKR1/AKR2*, an integral component of the membrane with palmitoyltransferase activity; *LEU2*, involved in leucine biosynthesis; *MRS3/MRS4*, iron transporter genes; *APE1*, with metalloaminopeptidase activity; and *AHP1*, a thiol-specific peroxiredoxin gene. The rest of these genes are upregulated, at both time points, and include the following: *TIS11/CTH1*, involved in mRNA processing; *CCC2*, a Cu^2+^-transporting P-type ATPase gene; *UBC8*, which negatively regulates gluconeogenesis; *ECL1*, which increases oxygen consumption and respiratory activity; *SMF3*, a putative divalent metal ion transporter gene; *ARA2*, an NAD-dependent arabinose dehydrogenase gene; *FET3*, a ferro-O_2_-oxidoreductase gene; and *PEP4*, a vacuolar protease gene. Most of these upregulated genes are related to ion transport and redox functions, in agreement with our GO term enrichment analysis.

### Multigene families significantly modulated upon lactic acid exposure.

We identified an unusual regulatory pattern in a family of genes related to S. cerevisiae *FDH1*, which codes for formate dehydrogenase. This enzyme is known to be induced upon formate exposure in S. cerevisiae and is widely found in methylotrophic yeasts (36, 37). Recent studies showed that the Fdh1 enzyme contributes to oxidative stress resistance in bacteria (38, 39). The Z. parabailii genome contains six genes in this family (*I01900*_*B*, *O01850*_*A*, *P02220*_*N*, *H05680*_*N*, *N02280*_*N*, and *F04070*_*N*), although formate dehydrogenase activity has not been demonstrated for any of them. All six *FDH*-like genes were highly upregulated at 18 h of lactic acid exposure. *P02220*_*N* is lactic acid specific (Table S2), and *N02280_N* and *F04070*_*N* were also significantly downregulated at 42 h (Data Set S5). Formate dehydrogenases perform the NAD^+^-dependent oxidation of formate to carbon dioxide. The S. cerevisiae strain CEN.PK 113-7D contains two *FDH* genes (*FDH1* and *FDH2*), whereas only *FDH1* is intact in the laboratory strain BY4741 because *FDH2* is truncated (37). The function of *FDH* genes in S. cerevisiae is not well characterized, but these enzymes have been better studied in methylotrophic yeasts such as Komagataella phaffi, where they are involved in the last step of the methanol dissimilation pathway (36).

**FIG 5:**
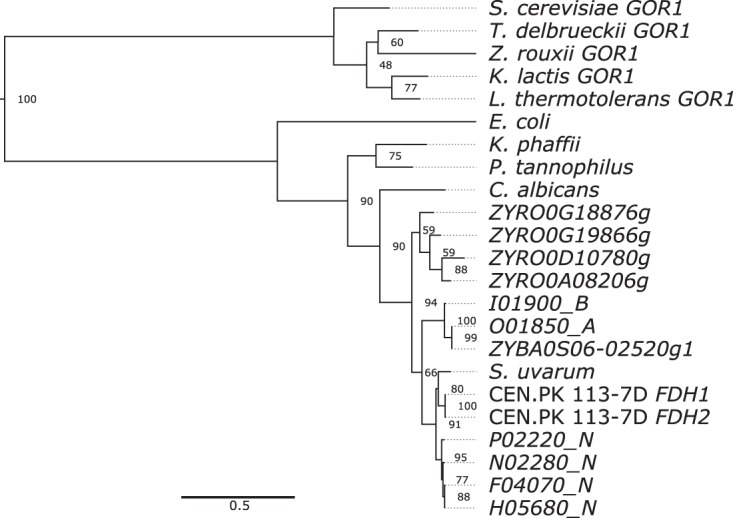
Phylogenetic tree of formate dehydrogenase amino acid sequences in yeast species (including Torulaspora delbrueckii, Zygosaccharomyces rouxii, Kluyveromyces lactis, Lachancea thermotolerans, Komagataella phaffii, Pachysolen tannophilus, Candida albicans, and Saccharomyces uvarum). The six Z. parabailii Fdh-like genes are shown (names ending in _A, _B, and _N). Prefixes ZYRO and ZYBA indicate genes from Z. rouxii and Z. bailii, respectively. The tree was rooted using the paralogous yeast gene *GOR1* (glyoxylate reductase), and Escherichia coli *FDH* is included for reference. The tree was constructed using PhyML. Bootstrap values from 100 replicates are shown.

Interestingly, the phylogenetic distribution of *FDH* genes among sequenced yeast genomes is rather patchy (36) and indicative both of recent gene amplifications and of multiple gene losses. We searched for *FDH* homologs in the NCBI databases and constructed a phylogenetic tree (Fig. 5). Many yeast species lack *FDH* genes completely, containing only homologs of distantly related genes such as *GOR1* (glyoxylate reductase). Nevertheless, the phylogenetic relationship among the *FDH* genes of the few species that retain this gene agrees well with the expected relationship among these species (Fig. 5). This observation suggests that the patchy distribution is due to numerous losses of an ancestral *FDH* gene (for example, in the genera Torulaspora, Lachancea, and Kluyveromyces) and not the result of horizontal gene transfer. There is essentially no conservation of synteny among the existing *FDH* genes, which shows that multiple species-specific gene duplications and gene relocations have occurred. Of the six Z. parabailii *FDH*-like genes, four are closely related and form a phylogenetic cluster with Saccharomyces species (Fig. 5). The other two form a cluster with the only *FDH*-like gene we identified in the genome of CLIB213^T^, a Z. bailii sensu stricto strain. The sister species Zygosaccharomyces rouxii has four *FDH*-like genes that cluster together in the tree. Thus, amplifications of *FDH*-like genes by gene duplication have occurred separately in Z. parabailii and Z. rouxii, and in the former species they are highly induced by lactic acid. This difference in *FDH* gene copy number between Z. parabailii and Z. bailii may be a contributory factor in the difference in their tolerance to lactic acid, as our previous study showed that Z. parabailii ATCC 60483 is more resilient to lactic acid than the *Z. bailii sensu stricto* strains ATCC 8766 and ATCC 58445^T^ (synonymous with CLIB213^T^) (20).

We searched systematically for other Z. parabailii genes assigned to multigene families which have significant expression changes in lactic acid. This was done by searching for sets of three or more Z. parabailii genes that share the same Z. bailii ortholog. We examined a total of 123 Z. parabailii genes in multigene families of this type, of which 22 are differentially expressed at at least one time point (classified as category 9 in Data Set S5). These 22 genes belong to 12 different multigene families significantly modulated in lactic acid. For example, *F06230*_*N*, *N00190*_*A*, and *O04100*_*A* are homologs of the *FFZ2* transporters, which are specific to Zygosaccharomyces species and able to transport fructose and glucose when overexpressed in S. cerevisiae (40, 41). In this family, *F06230*_*N* is upregulated in lactic acid at both time points, whereas *N00190*_*A* is upregulated only at 18 h and *O04100_A* did not show significant expression changes. Another interesting family is *A10020*_*N*, *G00240*_*N*, and *P00180*_*N*, which are all lactic acid specific (Table S2), significantly upregulated in lactic acid (when the log_2_-fold change cutoff is ignored), and homologous to the iron siderophore transporter gene *FIT2* putatively under the control of Aft1/Aft2 (Fig. 4B; Data Set S7). This family also includes *K00140*_*A* and *C00210*_*N*, for which we did not observe any evidence of expression. Furthermore, given that *K00140*_*A* is identical to the only *FIT2* homolog annotated in Z. bailii strain CLIB213^T^ (BN860_19394g1_1), and it is not differentially regulated, the Z. parabailii-specific genes in this family may have functional relevance.

## DISCUSSION

We aim to engineer a yeast strain able to produce lactic acid which, at high concentrations, is toxic to the cells. Our results indicate that, in general terms, Z. parabailii counteracts the toxicity of lactic acid by modulating its oxidation-reduction processes and the composition of its cell boundaries. Although some of these responses overlap those of S. cerevisiae, Z. parabailii additionally shows an interplay between its two homeologous gene sets and utilizes expanded multigene families. Validation of these observations awaits the development of better molecular tools for manipulation of Z. parabailii, but our work nevertheless represents a significant step toward engineering this nonconventional yeast to produce lactic acid.

The toxicity of lactic acid, and weak acids in general, involves dissipation of the pH gradient at the plasma membrane (42). Other secondary effects result from the intracellular accumulation of the weak acid counteranions. For example, acetate has been shown to trigger programmed cell death and an increase in the formation of reactive oxygen species (ROS) (12), while sorbate affects the membrane structure (43). Microorganisms have developed different mechanisms to tolerate these toxic effects. For example, S. cerevisiae responds to weak acids by using H^+^-ATPases to control intracellular pH (44). In Pichia anomala, a higher tolerance is achieved by coupling H^+^-ATPases with increased mitochondrial ATP production (45). Candida krusei also has higher tolerance to lactic and acetic acids than S. cerevisiae, postulated to involve a quicker H^+^-ATPase response (46). Z. parabailii shares this ATP-dependent tolerance response but has some unique features, connected to the hybrid nature of its genome, as discussed below.

We observed upregulation of genes related to detoxification of ROS which could be linked to the upregulation of the respiratory chain and the glyoxylate cycle. Lactic acid stress has been reported to imbalance the prooxidant/antioxidant ratio (47) and trigger the accumulation of ROS via the Fenton reaction (48). Accordingly, overexpression of cytosolic catalase or introduction of the pathway for biosynthesis of l-ascorbic acid (a well-known antioxidant) into S. cerevisiae improved resistance to oxidative and lactic acid stress (49, 50). The alleviating effect of antioxidants indicates the importance of controlling the concentration of H_2_O_2_, which can catalyze the conversion of glyoxylate into formate and CO_2_ (51). The upregulation of the glyoxylate cycle, combined with respiratory chain, would then result in the production of H_2_O_2_ and formate. The expansion and upregulation of the *FDH* multigene family in Z. parabailii would then serve to convert the otherwise toxic formate into NADH and CO_2_. This mechanism was described in the bacterium Pseudomonas fluorescens as an antioxidative defense mechanism (39, 52), and we speculate that the multiple Fdh enzymes in Z. parabailii might serve a similar role.

There are significant differences between the response to lactic acid that we observed in Z. parabailii and the responses previously reported in S. cerevisiae (32, 33). While many of these differences may reflect differences in physiology of the two yeasts, there were also differences in experimental setup used. We used microaerobic conditions, whereas previous studies used anaerobic chemostat conditions (32) and batch flask fermentation (33). Nevertheless, we also identified some similarities between the lactic acid responses in S. cerevisiae and Z. parabailii, involving iron homeostasis genes such as those encoding siderophore transporters and iron transporters. In S. cerevisiae, a high concentration of lactate ions in the growth medium chelates free iron, reducing its availability for cellular functions (32) and triggering a strong regulation of iron homeostasis (32, 33). We observed a similar response to lactic acid stress in Z. parabailii.

We found that Z. parabailii appears to modulate its cell wall in response to lactic acid stress. The cell wall is generally considered to be a barrier for large molecules (53, 54). Nevertheless, studies on S. cerevisiae have reported changes in expression of genes coding for cell wall components (55), or related to cell wall integrity (56), in response to acetic acid or to a low pH environment (57). In Z. parabailii, the downregulation of cell wall-related genes that we observed in this study can be linked to the decrease of cell wall mannoprotein and β 1→3 glucan levels that we previously found by Fourier transform infrared spectroscopy (FTIR) analysis (20). Together with the peculiar plasma membrane composition (19), these changes in the cell wall may contribute to the superior lactic acid tolerance of Z. parabailii.

The expression of Haa1-regulated genes during stress in Z. parabailii is rather different from that in S. cerevisiae. Haa1 has been reported to be a transcriptional activator of genes in response to both acetic acid and lactic acid in S. cerevisiae (32, 58, 59) and in response to acetic acid in Z. bailii (lactic acid was not investigated) (23, 60). It is intriguing to observe a different expression pattern for those genes in Z. parabailii during lactic acid stress, but further studies will be necessary to fully characterize the divergence of the roles of the Haa1 orthologs in the two species.

We found that lactic acid stress induces robust and statistically significant divergent expression responses between the homeologous gene pairs in Z. parabailii. These differences need to be further explored when considering differentially expressed genes as engineering targets, but the overall stress response we saw among them is striking. Homeologous gene pairs are present in all hybrid (allopolyploid) organisms (26). Most previous transcriptomic analyses involving homeologous pairs have been carried out in plant species (61–63), although there are examples with fungi (64) and yeasts (65, 66). We are not aware of any previous studies that found a similar genome-wide increase in homeolog expression divergence under stress conditions. Our study differs from previous work on yeast hybrids because we examined gene expression in a natural hybrid isolate, whereas preceding studies analyzed synthetic hybrids (65, 66). Furthermore, we compared expression between homeolog pairs under two different growth conditions, whereas previous comparisons were done against the parental genes (65, 66), even when more than one condition was used (65).

Our study is a pioneering approach to examining the transcriptome of a hybrid yeast. It was made possible by the availability of a high-quality reference genome sequence (25), which is often not available for other hybrid organisms. It also required highly stringent and tailored methods to measure the expression of highly similar genes and even identical copies. We showed that homeologous gene pairs have different expression patterns when subjected to acid stress, which could reflect or override transcriptional control mechanisms inherited from the parents of this hybrid. This hybrid nature is one of a few differences we observed in comparison with the lactic acid responses reported for S. cerevisiae and Z. bailii. Our observations need further experimental validation given that changes in transcript levels are not always reflected in protein activities *in vivo*. Nevertheless, our observations that the duplicated homologs of *DLD1* and *DLD2* are expressed only at a low level in lactic acid and that *DLD1* shows a statistically significant 2-fold decrease in expression in lactic acid are consistent with the absence of lactic acid consumption by *Z*. parabailii under these conditions (20), which is a key feature needed for a lactic acid-producing host. Our study provides methods and data to facilitate the understanding of molecular responses during acid stress in this or other hybrid yeasts, which is important for both fundamental and applied science.

## MATERIALS AND METHODS

### Cell growth, RNA extraction, and sequencing.

Z. parabailii strain ATCC 60483 was used for bioreactor fermentation. Cell aliquots, stored at −80°C in yeast extract-peptone-dextrose (YPD) glycerol stock, were grown to mid-exponential phase before being inoculated into the bioreactor at a final absorbance (optical density at 660 nm [OD_660_]) of 0.1. We used 2× Verduyn growth medium (67) at pH 3 containing 40 g liter^−1^ glucose with 40 g liter^−1^ lactic acid or no lactic acid (control condition). The fermentations were performed in 2-liter-volume bioreactors (Biostat B; Sartorius AG, Germany) with an operative volume of 1.5 liters. The temperature was maintained at 30°C, pH 3, by the addition of 4 M NaOH, and the stirrer speed was set to 400 rpm. The inlet gas flow was adjusted by two mass flow controllers (Bronkhornst high-tech EL-FLOW Select). The mass flow was set to obtain a mixture of N_2_ and air with a final concentration of inlet oxygen of 5%. The mixture was sparged at 0.75 vvm. Antifoam (Antifoam 204; Sigma-Aldrich) was used for foaming control.

The samples for RNA sequencing were taken in triplicate at 18 h and 42 h from the bioreactor fermentation, corresponding to log phase and postdiauxic shift, respectively (20). Total RNA was extracted using a Zymo Research Fungal/Bacterial RNA MiniPrep kit (Irvine, USA), and the quality of the RNA samples was evaluated with an Agilent bioanalyzer. The RNA samples were sequenced using the Illumina HiSeq2000 platform with 100-nucleotide (nt)-long paired-end reads at Parco Tecnologico Padano (Lodi, Italy).

### RNA-seq analysis.

We used our Z. parabailii ATCC 60483 genome annotation as a reference (25). This annotation consists of 10,072 nuclear and 13 mitochondrial protein-encoding genes obtained using an improved version of the Yeast Genome Annotation Pipeline (68) and includes additional metadata as an aid for functional interpretation. Briefly, because of its hybrid nature, the Z. parabailii genome contains two homeologous copies of most genes. We use suffixes _A and _B in gene names to indicate the two copies, where _A indicates gene copies that are virtually identical to their *Z. bailii sensu stricto* orthologs and _B indicates copies that are more divergent (5 to 25% synonymous sequence divergence). A few genes have the suffix _N because they could not be assigned to either of these two groups. There are 4,139 homeologous A:B gene pairs in the annotated genome sequence, but for 3 pairs we did not detect transcription of either of the genes under any condition, so these 3 pairs were not analyzed further.

Some extra information (see Data Set S1 in the supplemental material) was added to the original annotation, including functional domains and protein family memberships, which were obtained by aligning all the Z. parabailii ATCC 60483 amino acid sequences against the PFAM database (69) using HMMER v.3.0 (70). A genome-wide annotation of transmembrane proteins was also made by comparing the Z. parabailii proteome against the TransportDB 2.0 (71) database using BLAST v.2.2.22 (72). The sequences were then filtered based on identity (>35%) and coverage (>80%) and submitted to the TMHMM server v.2.0 (73) to determine a minimum of two potential transmembrane domains per sequence. Blast2GO (74) was then used to generate a custom gene ontology (GO) annotation for Z. parabailii (Data Set S2).

The raw RNA-seq reads were mapped against the Z. parabailii ATCC 60483 nuclear and mitochondrial genomes (25) using Bowtie v.1.1.2 (75) with the parameters -v 0 -k 10 --best -M 1. The parameter -v 0 gives high stringency by allowing no mismatches in the alignments discriminating between highly similar regions in the genome and discarding reads with sequencing artifacts. The parameters -k 10 --best -M 1 report only the best possible alignment out of up to 10 alternatives and, in case there are two equivalent best hits, only one is reported at random. This procedure reports multimapping reads with the tag “XM:i:2” and a mapping quality (MAPQ) equal to 0.

The mapped reads were subsequently counted using htseq-count v.0.6.0 (76) with two different settings. In the first case, htseq-count was applied to the full set of Z. parabailii genes with default parameters, to generate what we refer to as “full” counts. This setting discards the alignments for multimapping reads, because their quality is artificially set to the lowest possible value. Then, to obtain data from pairs of identical genes, a different htseq-count run was performed with the parameter -a 0 allowing for an MAPQ of ≥0. To avoid spurious low-quality alignments, this second run used only the alignments with the XM:i:2 tag and was applied only to a set of 232 duplicated genes that have 100% sequence identity (measured using blastn [72]), over their full length, to one or more other Z. parabailii genes. The counts from this second htseq-run for duplicated genes with multimapping reads are referred as “duplicated” counts. The duplicated counts represent a composite signal from two or more identical genes and potential different quality values, which is not the case for the “full” counts. Therefore, the two sets of counts were analyzed separately. All the counts reported are “full” counts unless stated otherwise.

The RNA-seq read counts were split into four groups according to condition and time point, each group containing three libraries. One of the libraries for the control condition at 18 h contained few reads (5.9 million, compared to the average of 30.5 million from the other libraries) and was excluded from further analyses. We therefore used the TMM method (77) implemented in edgeR v.3.18.1 (78) to normalize the read counts and provide better comparability across the different-sized samples. Counts per million (CPM) were calculated from the normalized counts using edgeR. Genes with less than 1 CPM in at least three samples from the same condition were considered to have no evidence of expression. We also calculated reads per kilobase of transcript per million mapped reads (RPKM) using edgeR. This was done for the normalized and filtered sets of “full” counts for 4,136 homeolog pairs and for the “duplicated” counts for the 232 duplicated genes.

An expression ratio index was calculated for the 4,136 A:B homeolog pairs for which at least one gene in the pair showed evidence of expression. There are 4,139 homeologous A:B gene pairs in the annotated genome sequence, but for 3 pairs we did not detect transcription of either of the genes under any condition, so these 3 pairs were not analyzed further. The expression ratio is calculated as follows: expression ratio = avg RPKM_A_*/*(avg RPKM_A_ *+* avg RPKM_B_) where the subscripts A and B indicate the parental origin of each gene and avg is the average. This index ranges from 0 to 1, with 0.5 meaning equal expression of the A and B homeologs. RPKM values for each homeolog in a pair, averaged among replicates, are given in Data Set S3. We calculated descriptive statistics from the expression ratio for the different groups using the R package psych v.1.7.5 (79). Exact binomial tests and Fligner-Killeen tests were performed using the R functions binom.test and fligner.test. The R function p.adjust was used for Bonferroni's correction of *P* values for multiple testing.

The normalized and filtered data sets were Voom transformed (80) to consider the differences in count sizes (or sequencing depth) and the overall data set variability. This was followed by differential expression analysis (DEA) with an adjusted *P* value of <0.05 and a ∣log_2_-fold change∣ of ≥1 for statistical significance (Data Set S4 for the “full” set; Data Set S5 for the “duplicated” set). Both the Voom transformation and the differential expression analysis were done using Limma v.3.32.2 (81). The Z. parabailii GO annotation was utilized for GO term enrichment analysis with the R package goseq v.1.28.0 (82), applied to the three sets of upregulated genes (18 h, 42 h, and both time points) as well as to the corresponding three sets of downregulated genes (Data Set S6). The output of goseq was visualized using UpsetR v.1.3.3 (83).

### Phylogenetic analysis of formate dehydrogenase sequences.

Protein sequences of homologs of the Z. parabailii Fdh-like proteins were identified by blastp (72) searches against the nonredundant protein sequence database of the National Center for Biotechnology Information (NCBI) with default parameters. The search was restricted to yeast species (taxid:4932). Representative sequences were selected from the species indicated in Fig. 5. For yeast species that lacked an apparent Fdh, we retained the next-most-similar protein instead, which in all cases had higher similarity to S. cerevisiae Gor1, another protein with an NAD(P)-binding domain. Escherichia coli Fdh1, which is more closely related to yeast Fdh1 than to yeast Gor1, was included for reference. Alignments and phylogenetic trees were generated in SeaView v.4 (84) with the included versions of Clustal Omega (85) and PhyML (86) using 100 bootstrap replicates. The output was visualized using FigTree v.1.4.3 (http://tree.bio.ed.ac.uk/software/figtree/).

### Accession number(s).

The RNA-seq data reported here have been deposited in NCBI's Gene Expression Omnibus (87) under accession number GSE104654. The submitted data include the RNA-seq fastq files, counts for the “full” and “duplicated” sets (both raw and normalized), and RPKM values for the duplicated genes.

## Supplementary Material

Supplemental material
