# Supplementary material for: Transcriptional Response to Lactic Acid Stress in the Hybrid Yeast Zygosaccharomyces parabailii
Source: Appl Environ Microbiol. 2018 Feb 14;84(5):e02294-17. doi: 10.1128/AEM.02294-17 (PMC5812937; doi:10.1128/AEM.02294-17)
Supplement: Supplemental material [file supp_84_5_e02294-17__index.html]

Supplemental material 

# Transcriptional Response to Lactic Acid Stress in the Hybrid Yeast Zygosaccharomyces parabailii

## Supplemental material

- Supplemental file 1 -

  Control-specific genes (Table S1); lactic acid-specific genes (Table S2); data set descriptions.

  PDF, 116K
- Supplemental file 2 -

  Full annotation table for the *Z. parabailii* genome (Data Set S1).

  XLSX, 940K
- Supplemental file 3 -

  *Z. parabailii* functional annotation (Data Set S2).

  XLSX, 1.0M
- Supplemental file 4 -

  Averaged reads per kilobase of transcript per million mapped reads for homeolog gene pairs (Data Set S3).

  XLSX, 489K
- Supplemental file 5 -

  Duplicated genes differentially expressed during lactic acid exposure (Data Set S4).

  XLSX, 88K
- Supplemental file 6 -

  Differentially expressed genes during lactic acid exposure (Data Set S5).

  XLSX, 283K
- Supplemental file 7 -

  Enriched GO terms among differentially expressed genes (Data Set S6).

  XLSX, 19K
- Supplemental file 8 -

  List of differentially expressed genes controlled by Haa1 and Aft1/2 (Data Set S7).

  XLSX, 24K
