# Supplementary material for: Transcriptional Response to Lactic Acid Stress in the Hybrid Yeast Zygosaccharomyces parabailii
Source: Appl Environ Microbiol. 2018 Feb 14;84(5):e02294-17. doi: 10.1128/AEM.02294-17 (PMC5812937; doi:10.1128/AEM.02294-17)
Supplement: Supplemental material [file AEM.02294-17_zam005188353s1.pdf]

Supplemental Material

Transcriptional response to lactic acid stress in the hybrid yeast *Zygosaccharomyces parabailii*

Raúl A. Ortiz-Merino[a], Nurzhan Kuanyshev[b,c], Kevin P. Byrne[a], Javier A. Varela[c], John P. Morrissey[c], Danilo Porro[b], Kenneth H. Wolfe[a], Paola Branduardi[b]#

UCD Conway Institute, School of Medicine, University College Dublin, Dublin, Ireland[a;] Department of Biotechnology and Biosciences, University of Milano-Bicocca, Milano, Italy[b]; School of Microbiology/Centre for Synthetic Biology and Biotechnology/Environmental Research Institute/APC Microbiome Institute, University College Cork, Cork, Ireland[c]

#Address correspondence to Paola Branduardi, paola.branduardi@unimib.it

Table S1. Control-specific genes.

ID	NCBI_ID	Description
A00140_N	BZL39_A00140	related to L-lactate dehydrogenase
A01200_B	BZL39_A01200	uncharacterized protein ZBAI_04890
A02950_B	BZL39_A02950	uncharacterized protein ZBAI_04711
A04460_B	BZL39_A04460	related to PCH2- ATPase
A05220_N	BZL39_A05220	uncharacterized protein ZBAI_04496
A09510_B	BZL39_A09510	related to IRC11-Strong similarity to YDR391c
A09995_N	BZL39_A09995	---NA---
A10080_N	BZL39_A10080	ZYBA0S13-00232g1_1
B03300_A	BZL39_B03300	ZYBA0S04-07030g1_1
B09380_A	BZL39_B09380	A Chain Crystal Structure Of Yth Domain Of Zygosaccharomyces rouxii Mrb1 In Complex With N6-methyladenosine RNA
B09470_A	BZL39_B09470	related to IRC11-Strong similarity to YDR391c
B09730_A	BZL39_B09730	ZYBA0S13-00980g1_1
B10130_N	BZL39_B10130	CIC11C00000000719
C03800_A	BZL39_C03800	BN860_11408g1_1
D01770_B	BZL39_D01770	uncharacterized protein ZBAI_02445
E02670_A	BZL39_E02670	BN860_10704g1_1
E07220_N	BZL39_E07220	Y element ATP-dependent helicase
G06420_N	BZL39_G06420	related to ketopantoate reductase
H01670_B	BZL39_H01670	uncharacterized protein ZBAI_06090
H04800_B	BZL39_H04800	uncharacterized protein ZBAI_05789
H06370_A	BZL39_H06370	related to ornithine aminotransferase
I04800_A	BZL39_I04800	related to Peroxisomal membrane PAS20
J00180_N	BZL39_J00180	related to ornithine aminotransferase
J03940_A	BZL39_J03940	cell wall CWP1
K04960_A	BZL39_K04960	probable OPT1-High-affinity glutathione transporter
K05090_N	BZL39_K05090	related to permease of the major facilitator superfamily
M00100_N	BZL39_M00100	Y element ATP-dependent helicase
M00110_N	BZL39_M00110	Y element ATP-dependent helicase 1 copy 6
M00290_N	BZL39_M00290	related to ornithine aminotransferase
N00210_N	BZL39_N00210	related to MFS multidrug transporter
N05030_A	BZL39_N05030	related to Peroxisomal membrane PAS20

Table S2. Lactic acid-specific genes.

ID	NCBI_ID	Description
A00170_N	BZL39_A00170	threonine dehydratase
A00180_A	BZL39_A00180	probable Allantoate permease
A00210_B	BZL39_A00210	probable Constitutive acid phosphatase
A00290_A	BZL39_A00290	related to thermophilic desulfurizing enzyme
A05200_N	BZL39_A05200	uncharacterized protein ZBAI_04498
A07530_B	BZL39_A07530	uncharacterized protein ZBAI_04271
A09010_N	BZL39_A09010	uncharacterized protein ZBAI_08640
A10020_N	BZL39_A10020	Fit2p
A10040_B	BZL39_A10040	related to Siderophore iron transporter 3
B00200_A	BZL39_B00200	related to ketopantoate reductase
B10000_A	BZL39_B10000	related to Siderophore iron transporter 3
C00430_A	BZL39_C00430	BN860_18976g1_1
C05100_A	BZL39_C05100	BN860_08460g1_1
E00130_A	BZL39_E00130	BN860_16600g1_1
E01370_A	BZL39_E01370	related to Similarity to YIL123w and Sun4p
E02440_A	BZL39_E02440	BN860_11232g1_1
E06720_A	BZL39_E06720	BN860_01398g1_1
F06250_N	BZL39_F06250	probable Vacuolar sorting targeting 10
G00240_N	BZL39_G00240	Fit2p
G01010_A	BZL39_G01010	Gor1p
J00140_N	BZL39_J00140	1-aminocyclopropane-1-carboxylate deaminase
J05500_A	BZL39_J05500	ZYBA0S03-00892g1_1
K00230_N	BZL39_K00230	uncharacterized protein ZBAI_09841
K05040_N	BZL39_K05040	ZYBA0S13-00144g1_1
K05170_A	BZL39_K05170	related to ketopantoate reductase
L05300_N	BZL39_L05300	related to Soluble epoxide hydrolase
M00150_N	BZL39_M00150	uncharacterized protein ZBAI_01365
M00180_N	BZL39_M00180	uncharacterized protein ZBAI_01367
M00220_N	BZL39_M00220	related to MFS multidrug transporter
N00200_N	BZL39_N00200	Hxt3p
N00290_A	BZL39_N00290	probable Fluconazole resistance 1
O04150_N	BZL39_O04150	uncharacterized protein ZBAI_08167
O04170_A	BZL39_O04170	related to Arsenical-resistance 3
P00180_N	BZL39_P00180	Fit2p
P02220_N	BZL39_P02220	Fdh1p
ZPARMT0022	BZL39_MT0022	NA

Supplemental file 2: **Data Set S1.** Full annotation table for the *Z. parabailii* genome. The table includes the *Z. parabailii* gene codes, their corresponding *Z. bailii* homeolog (if any), the *S. cerevisiae* homolog(s) (if any), and the gene category as reported in Ortiz-Merino *et al*. 2017 (1). It also includes the PFAM (2) category and a logical column TM indicating if the gene has more than 2 transmembrane domains and has hits in TransportDB (3) as described in the method section. The names and systematic IDs for the corresponding *S. cerevisiae* homologs assigned by YGAP (4) are also included with a link to their description in the Saccharomyces Genome Database (http://www.yeastgenome.org).

Supplemental file 3: **Data Set S2.** *Z. parabailii* functional annotation obtained using Blast2GO (5). The table includes a brief description of the best blast (6) hit including the number of hits and e-Value found by Blast2GO as described in the software documentation. In addition, it shows the assigned Gene Ontology terms and Enzyme Classification codes with a brief description.

Supplemental file 4: **Data Set S3.** Averaged Reads Per Kilobase of transcript per Million mapped reads (RPKM) values for homeolog gene pairs. Columns are labels as C or LA corresponding to control or lactic acid and 18 or 42 corresponding to the timepoint. Columns with the suffix _A correspond to the A gene similar to _B and B genes.

Supplemental file 5: **Data Set S4.** Duplicated genes differentially expressed during lactic acid exposure. In separate sheets, the *Z. parabailii* duplicated genes found as up- or downregulated in the different time points. Each sheet includes the corresponding statistical values obtained for each gene form the Limma-Voom analysis (7, 8) and the functional metadata described in the methods section.

Supplemental file 6: **Data Set S5.** Differentially expressed genes during lactic acid exposure. In separate sheets, the *Z. parabailii* genes found as up- or downregulated in the different time points. Each sheet includes the corresponding statistical values obtained for each gene form the Limma-Voom (7, 8) analysis and the functional metadata described in the methods section. Enriched GO terms amongst the differentially expressed genes. In separate sheets, the terms found as enriched with an adjusted P value ≤ 0.05 using goseq (9) for the upregulated and downregulated gene sets. The value was $\log_{10}$ transformed to facilitate visual interpretation and it is shown when obtained at each individual time point or both.

Supplemental file 7: **Data Set S6.** Enriched GO terms among differentially expressed genes. In separate sheets, the terms found as enriched with an adjusted P value ≤ 0.05 using goseq (9) for the upregulated and downregulated gene sets. The value was $\log_{10}$ transformed to facilitate visual interpretation and it is shown when obtained at each individual time point or both.

Supplemental file 8: **Data Set S7.** List of differentially expressed genes controlled by Haa1 and Aft1/2. *Z. parabailii* genes whose *S. cerevisiae* homolog is under control of either Haa1 or Aft1/2 according to YEASTRACT (10). The $\log_2$-fold change values are shown in the case they were found as differentially expressed at 18 h or 42 h. The *S. cerevisiae* standard or systematic names are shown including the corresponding paralog if there is any.

References

1. **Ortiz-Merino RA, Kuanyshev N, Braun-Galleani S, Byrne KP, Porro D, Branduardi P, Wolfe KH.** 2017. Evolutionary restoration of fertility in an interspecies hybrid yeast, by whole-genome duplication after a failed mating-type switch. PLoS Biol **15:**e2002128.
2. **Finn RD, Bateman A, Clements J, Coggill P, Eberhardt RY, Eddy SR, Heger A, Hetherington K, Holm L, Mistry J, Sonnhammer EL, Tate J, Punta M.** 2014. Pfam: the protein families database. Nucleic Acids Res **42:**D222-230.
3. **Elbourne LD, Tetu SG, Hassan KA, Paulsen IT.** 2017. TransportDB 2.0: a database for exploring membrane transporters in sequenced genomes from all domains of life. Nucleic Acids Res **45:**D320-D324.
4. **Proux-Wera E, Armisen D, Byrne KP, Wolfe KH.** 2012. A pipeline for automated annotation of yeast genome sequences by a conserved-synteny approach. BMC Bioinformatics **13:**237.
5. **Gotz S, Garcia-Gomez JM, Terol J, Williams TD, Nagaraj SH, Nueda MJ, Robles M, Talon M, Dopazo J, Conesa A.** 2008. High-throughput functional annotation and data mining with the Blast2GO suite. Nucleic Acids Res **36:**3420-3435.
6. **Altschul SF, Gish W, Miller W, Myers EW, Lipman DJ.** 1990. Basic local alignment search tool. J Mol Biol **215:**403-410.
7. **Ritchie ME, Phipson B, Wu D, Hu Y, Law CW, Shi W, Smyth GK.** 2015. limma powers differential expression analyses for RNA-sequencing and microarray studies. Nucleic Acids Res **43:**e47.
8. **Law CW, Chen Y, Shi W, Smyth GK.** 2014. voom: Precision weights unlock linear model analysis tools for RNA-seq read counts. Genome Biol **15:**R29.
9. **Young MD, Wakefield MJ, Smyth GK, Oshlack A.** 2010. Gene ontology analysis for RNA-seq: accounting for selection bias. Genome Biol **11:**R14.
10. **Teixeira MC, Monteiro P, Jain P, Tenreiro S, Fernandes AR, Mira NP, Alenquer M, Freitas AT, Oliveira AL, Sa-Correia I.** 2006. The YEASTRACT database: a tool for the analysis of transcription regulatory associations in Saccharomyces cerevisiae. Nucleic Acids Res **34:**D446-451.
